# Enhanced hydrogen generation by reverse spillover effects over bicomponent catalysts

**DOI:** 10.1038/s41467-021-27785-5

**Published:** 2022-01-10

**Authors:** Zhe Gao, Guofu Wang, Tingyu Lei, Zhengxing Lv, Mi Xiong, Liancheng Wang, Shuangfeng Xing, Jingyuan Ma, Zheng Jiang, Yong Qin

**Affiliations:** 1 State Key Laboratory of Coal Conversion, Institute of Coal Chemistry, Chinese Academy of Sciences, Taiyuan, 030001 China; 2 Center of Materials Science and Optoelectronics Engineering, University of Chinese Academy of Sciences, Beijing, 100049 China; 3 Shanghai Synchrotron Radiation Facility, Shanghai Advanced Research Institute, Chinese Academy of Sciences, Shanghai, 201204 China

**Keywords:** Catalytic mechanisms, Heterogeneous catalysis, Chemical engineering

## Abstract

The contribution of the reverse spillover effect to hydrogen generation reactions is still controversial. Herein, the promotion functions for reverse spillover in the ammonia borane hydrolysis reaction are proven by constructing a spatially separated NiO/Al_2_O_3_/Pt bicomponent catalyst via atomic layer deposition and performing in situ quick X-ray absorption near-edge structure (XANES) characterization. For the NiO/Al_2_O_3_/Pt catalyst, NiO and Pt nanoparticles are attached to the outer and inner surfaces of Al_2_O_3_ nanotubes, respectively. In situ XANES results reveal that for ammonia borane hydrolysis, the H species generated at NiO sites spill across the support to the Pt sites reversely. The reverse spillover effects account for enhanced H_2_ generation rates for NiO/Al_2_O_3_/Pt. For the CoO_*x*_/Al_2_O_3_/Pt and NiO/TiO_2_/Pt catalysts, reverse spillover effects are also confirmed. We believe that an in-depth understanding of the reverse effects will be helpful to clarify the catalytic mechanisms and provide a guide for designing highly efficient catalysts for hydrogen generation reactions.

## Abstract (web summary)

H_2_ is regarded as an attractive green fuel and a promising energy carrier for the future. Here, the promotion functions of reverse spillover effects in the H_2_ generation reactions over bicomponent catalysts are revealed by performing in situ X ray absorption near edge structure characterization.

## Introduction

The ever-increasing global energy demand and the detrimental effect of the CO_2_ product of fossil fuels have triggered a widespread search for alternative energy sources, which are effective and renewable and do not cause further environmental issues^1^. Because of its high energy density and renewability, H_2_ has been regarded as an attractive green fuel and a promising energy carrier for the future to meet increasing energy and environmental challenges^2^. Catalytic H_2_ generation from hydrogen storage materials is considered a potential method of H_2_ production if they can be effectively catalyzed^3,4^. The search for efficient catalytic systems would be greatly facilitated by a clearer understanding of the underlying chemical process.

Noble metal catalysts, such as Pt, Pd, and Ru, have been recognized as important classes of catalysts for hydrogen generation, due to their high catalytic activity and durability^5–11^. It is noted that coupling metal catalysts with secondary metals^12–15^ and/or transition metal oxides^16–25^ is an encouraging strategy to further enhance catalytic performance. In the past, various theories (e.g., the metal-oxide interfacial sites, electron interactions, or hydrogen reverse spillover effect) have been offered to explain the enhancement of H_2_ generation when different components are combined in a catalyst. For example, Francisco Zaera and coworkers argued that in the photocatalytic production of H_2_ from water with semiconductor catalysts, the role of metal additives is a reverse spillover effect, not to trap excited electrons^26^. Hydrogen reverse spillover, as a form of spillover, involves the migration of adsorbed hydrogen atoms from an oxide (or other nonmetal surface) to a metal, where they recombine to molecular hydrogen^27–29^. However, due to the lack of well-defined catalysts with clearly separated functional components and the difficulties in performing in situ characterization technologies, researchers have not formed an agreement on the enhancement mechanism. It is still a challenging issue to reveal the promotion effects of reverse spillover in H_2_ generation reactions.

In this work, taking the ammonia borane (NH_3_·BH_3_, AB) hydrolysis reaction as an example, the promotion functions of reverse spillover in this reaction are proven using a spatially separated NiO/Al_2_O_3_/Pt catalyst as a model catalyst, in combination with in situ quick XANES characterization. The NiO/Al_2_O_3_/Pt catalyst was prepared by a facile and general template-assisted atomic layer deposition (ALD) method^30–36^. In situ XANES results clearly reveal that for H_2_ generation from AB, the H species generated at NiO sites spill across the support to the Pt sites, i.e., reverse spillover phenomenon. This accounts for the enhanced H_2_ generation rates of bicomponent oxide-metal catalysts, compared with single component Pt-based catalysts. The reverse spillover effects are also confirmed for the CoO_*x*_/Al_2_O_3_/Pt and NiO/TiO_2_/Pt catalysts. Our study provides a guide for designing highly efficient catalysts for hydrogen generation reactions.

## Results and discussion

### Synthesis and characterization of the catalysts

The NiO/Al_2_O_3_/Pt catalyst was synthesized by ALD using carbon nanocoils (CNCs) as templates (Supplementary Fig. 1). First, Pt nanoparticles (20 ALD cycles) and an Al_2_O_3_ film (50 ALD cycles) were deposited onto CNCs. The CNC templates were then removed by calcination. Finally, NiO nanoparticles (100 ALD cycles) were deposited, obtaining NiO/Al_2_O_3_/Pt. Al_2_O_3_/Pt and NiO/Al_2_O_3_ were also produced as reference catalysts.

**Fig. 1:**
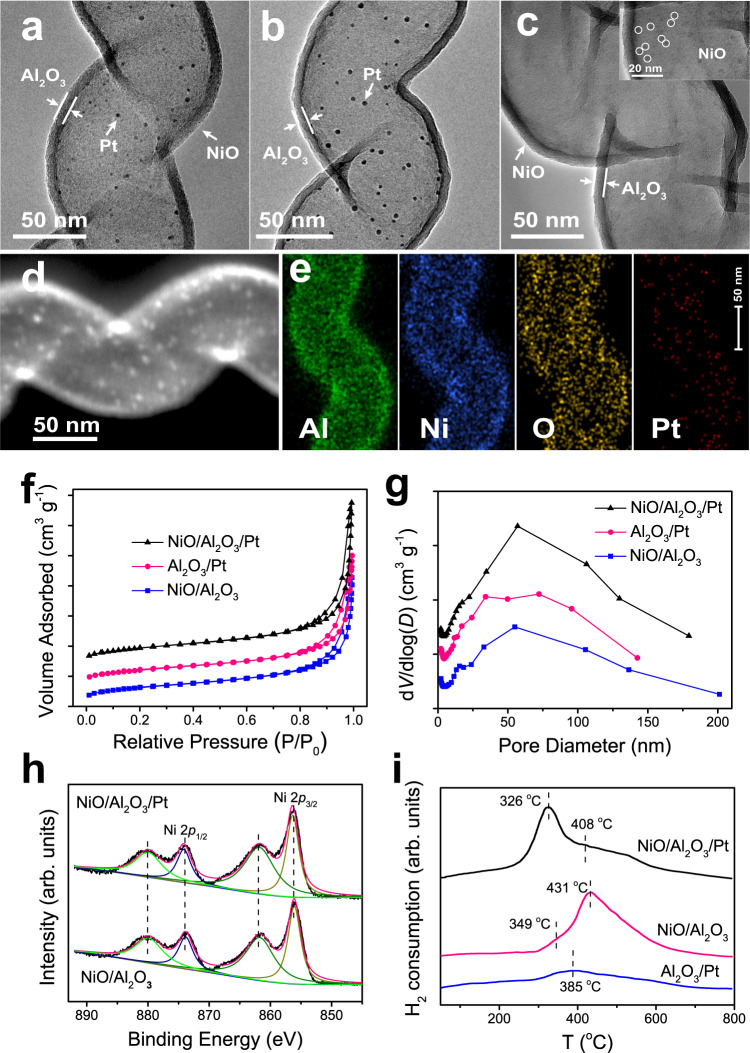
Structure and chemisorption characterization of the catalysts. TEM images of **a** NiO/Al_2_O_3_/Pt, **b** Al_2_O_3_/Pt, and **c** NiO/Al_2_O_3_ catalysts. Inset in **c** shows a HRTEM image of NiO/Al_2_O_3_. **d** HAADF-STEM image and **e** EDX elemental mapping of the NiO/Al_2_O_3_/Pt catalyst. **f** N_2_ adsorption−desorption isotherms and **g** the corresponding pore size distributions of the catalysts. **h** XPS Ni 2*p* analysis of NiO/Al_2_O_3_ and NiO/Al_2_O_3_/Pt. **i** H_2_-TPR profiles of NiO/Al_2_O_3_, Al_2_O_3_/Pt, and NiO/Al_2_O_3_/Pt.

Figure 1a shows transmission electron microscopy (TEM) image of NiO/Al_2_O_3_/Pt. Hollow Al_2_O_3_ nanotubes with a uniform wall thickness (ca. 7 nm) can be clearly observed. The lattice distance of Pt nanoparticles was measured to be ~0.226 nm (Supplementary Fig. 2), which corresponds to the Pt(111) plane. High-angle annular dark field scanning transmission electron microscopy (HAADF-STEM) image and energy-dispersive X-ray spectroscopy (EDX) mapping (Fig. 1d, e) for NiO/Al_2_O_3_/Pt show that Ni and Pt are distributed on the outer and inner surfaces of Al_2_O_3_ nanotubes, respectively. The STEM image, EDX mapping, and line-scanning profile for a cross-sectional specimen prepared by focused ion beam milling along the vertical direction of the Al_2_O_3_ nanotubes (Supplementary Fig. 3) further demonstrate the separated structure of NiO/Al_2_O_3_/Pt. TEM images of the Al_2_O_3_/Pt and NiO/Al_2_O_3_ catalysts are shown in Fig. 1b, c. There are no Pt particles on the outer surfaces of Al_2_O_3_ nanotubes for Al_2_O_3_/Pt (Supplementary Fig. 4). Due to the small size and low contrast of NiO nanoparticles, it is not straightforward to distinguish NiO nanoparticles in Fig. 1a, c. From the HRTEM image of NiO/Al_2_O_3_ (inset in Fig. 1c), NiO nanoparticles can be clearly observed. The Pt content in the catalysts was measured using inductively coupled plasma-atomic emission spectrometry (ICP-AES) to be 3.65 and 4.23% for NiO/Al_2_O_3_/Pt and Al_2_O_3_/Pt, and the Ni content was measured to be 8.05 and 8.71% for NiO/Al_2_O_3_/Pt and NiO/Al_2_O_3_, respectively. The N_2_ sorption isotherms for the NiO/Al_2_O_3_, Al_2_O_3_/Pt, and NiO/Al_2_O_3_/Pt catalysts almost overlap (Fig. 1f). The Brunauer–Emmett–Teller (BET) surface areas for the NiO/Al_2_O_3_, Al_2_O_3_/Pt, and NiO/Al_2_O_3_/Pt catalysts were calculated to be 95.4, 93.6, and 98.0 m^2^ g^–1^, respectively. Their pore volumes were 0.34, 0.34, and 0.39 cm^3^ g^–1^, respectively. The Barrett–Joiner–Halenda (BJH) pore size distribution curves (Fig. 1g) deduced from desorption branches of the N_2_ sorption isotherms confirm that NiO/Al_2_O_3_, Al_2_O_3_/Pt, and NiO/Al_2_O_3_/Pt samples are made up of pores with average sizes centred at 54.9, 54.4, and 57.0 nm, respectively. The pore sizes of these catalysts, i.e., the inner diameters of the Al_2_O_3_ nanotubes, correspond to the diameters of the CNC sacrificial templates. These results show that all the catalysts possess similar pore structures.

The X-ray photoelectron spectroscopy (XPS) results reveal the existence of Ni^2+^ species in NiO/Al_2_O_3_ and NiO/Al_2_O_3_/Pt (Fig. 1h). The XPS peaks for the two catalysts are similar. The XPS peaks located at binding energies of 856.1 and 874.0 eV are attributed to Ni 2*p*_3/2_ and Ni 2*p*_1/2_, respectively, and the peaks located at binding energies of 861.8 and 879.7 eV are attributed to satellite peaks. From the X-ray diffraction (XRD) patterns for the Al_2_O_3_/Pt and NiO/Al_2_O_3_/Pt catalysts (Supplementary Fig. 5), the presence of Pt nanoparticles can be confirmed. No diffraction peak assigned to NiO is detected from the XRD patterns for NiO/Al_2_O_3_ and NiO/Al_2_O_3_/Pt, which can be ascribed to the high dispersion of ALD-prepared nanoparticles. Hydrogen temperature programmed reduction (H_2_-TPR) was used to study the redox properties of the catalysts (Fig. 1i). The profile obtained for Al_2_O_3_/Pt displays a principal reduction peak at 385 °C, which can be attributed to Pt interacting with Al_2_O_3_^37^. The NiO/Al_2_O_3_ catalyst exhibits a small shoulder peak at approximately 349 °C and a strong peak centred at 431 °C, corresponding to the reductions of bulk NiO and the NiO interacting with Al_2_O_3_. In contrast, for NiO/Al_2_O_3_/Pt, the first H_2_ consumption peak (corresponding to the reduction of bulk NiO) shifts from 349 to 326 °C and becomes obvious, and a broadened peak centred at 408 °C (corresponding to the reductions of Pt and NiO interacting with Al_2_O_3_) can be observed. Quantification of the H_2_-TPR curves (Supplementary Table 1) shows that the hydrogen consumed by NiO/Al_2_O_3_/Pt (1.86 mmol H_2_ g^–1^) is greater than the sum of the hydrogen consumed by Al_2_O_3_/Pt (0.31 mmol H_2_ g^–1^) and NiO/Al_2_O_3_ (1.29 mmol H_2_ g^–1^). These results demonstrate that the reduction of NiO species is promoted after Pt addition, which can be attributed to the hydrogen spillover effect^38–41^. This hydrogen spillover effect is further confirmed from the results of in situ quick XANES experiments under a H_2_ atmosphere (0.6 MPa, 80 °C) (Supplementary Figs. 6, 7 and Table 2).

### Enhanced hydrogen generation after NiO addition

**Fig. 2:**
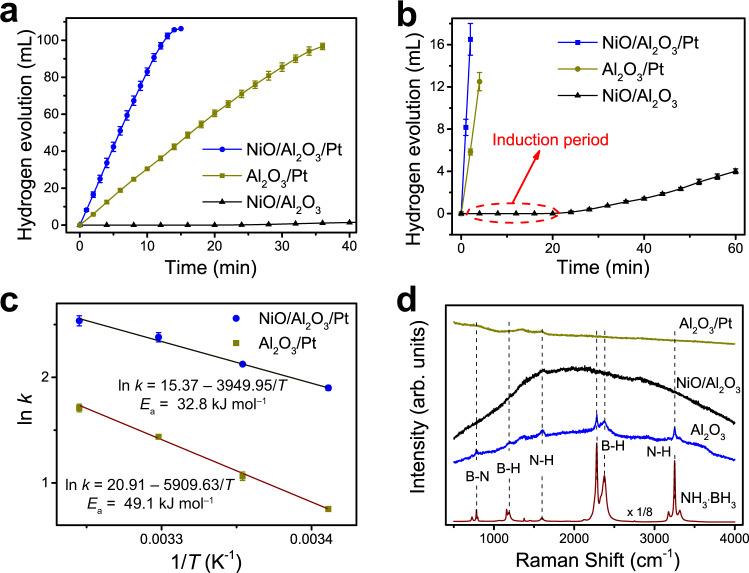
Catalytic performances of catalysts for the dehydrogenation reaction of AB and Raman spectra for the used catalysts. **a** Volume of H_2_ generated from AB solution (0.15 mol L^–1^) at 25 °C catalyzed by NiO/Al_2_O_3_, Al_2_O_3_/Pt, and NiO/Al_2_O_3_/Pt. **b** The volume of H_2_ generated at less than 18 mL versus prolonged time (60 min) clearly shows the induction period for NiO/Al_2_O_3_. **c** The Arrhenius plots for Al_2_O_3_/Pt and NiO/Al_2_O_3_/Pt. Error bars correspond to the standard deviation of three independent measurements. **d** Raman spectra for the used Al_2_O_3_ support, NiO/Al_2_O_3_, and Al_2_O_3_/Pt after reaction for 10 min and data for the reference sample NH_3_·BH_3_.

Here, the hydrolytic dehydrogenation of AB for H_2_ production is selected as a model reaction to investigate the reverse spillover effect. The catalytic performances of different catalysts for the dehydrogenation reaction of AB are shown in Fig. 2. A nearly linear H_2_ evolution curve is obtained for Al_2_O_3_/Pt, suggesting a zero-order reaction with respect to AB (Fig. 2a). For the NiO/Al_2_O_3_ catalyst, its H_2_ evolution curve exhibits a long induction period of approximately 20 min, after which the curve starts to rise gradually. The NiO particles for NiO/Al_2_O_3_ are located on the outer surfaces of the Al_2_O_3_ nanotubes. The reactant molecules (H_2_O and AB) easily access the exposed NiO sites. Thus, the mass transfer in the porous structures is unlikely to lead to the induction period of the NiO/Al_2_O_3_ catalyst. It is generally believed that new active species are generated during the induction period (Fig. 2b)^42–44^. However, even after reaction for 60 min, the H_2_ evolution volume is still only 3.8 mL, which shows an extremely poor activity for NiO/Al_2_O_3_. For NiO/Al_2_O_3_/Pt, a rapid and linear H_2_ evolution curve without an induction period is obtained. The time required to complete the hydrolysis reaction for the NiO/Al_2_O_3_/Pt catalyst is less than that for Al_2_O_3_/Pt, indicating that NiO addition can greatly enhance the activity of the Al_2_O_3_/Pt catalyst, even though NiO alone has little activity.

Kinetic experiments for the Al_2_O_3_/Pt and NiO/Al_2_O_3_/Pt catalysts were also conducted. The time dependences for H_2_ generation at various temperatures (20−35 °C) were recorded (Supplementary Fig. 8). The catalysts still retain a linear increase in the H_2_ generation volume with reaction time. According to Arrhenius plots of ln *k* versus 1/*T* (Fig. 2c), the activation energies (*E*_a_) for the hydrolysis of AB using Al_2_O_3_/Pt and NiO/Al_2_O_3_/Pt are calculated to be 49.1 and 32.8 kJ mol^–1^, respectively. The effect of the AB amount on the hydrolysis of AB was investigated (Supplementary Fig. 9). A nearly horizontal relationship in logarithmic plots between the H_2_ generation rate and AB concentration is further normalized, indicating that hydrolysis over NiO/Al_2_O_3_/Pt is also a zero-order reaction with respect to the AB concentration.

The effects of the distance between NiO and Pt components (i.e., the thicknesses of the Al_2_O_3_ support) and NiO loadings in the catalyst on the catalytic performance were investigated (Supplementary Fig. 10). The synthesis procedures for NiO/100Al_2_O_3_/Pt, NiO/200Al_2_O_3_/Pt, and 50NiO/Al_2_O_3_/Pt are similar to that for NiO/Al_2_O_3_/Pt, except for adjustment of the ALD cycles for the Al_2_O_3_ film and NiO nanoparticles (Supplementary Fig. 11). As expected, when the thickness of the Al_2_O_3_ support increases from 7 nm (NiO/Al_2_O_3_/Pt, 50 cycles of Al_2_O_3_) to 13 nm (NiO/100Al_2_O_3_/Pt, 100 cycles of Al_2_O_3_) and 25 nm (NiO/200Al_2_O_3_/Pt, 200 cycles of Al_2_O_3_), the catalytic activity decreases. As the NiO loading decreases, the H_2_ evolution rate also decreases. Even so, the activity of NiO/100Al_2_O_3_/Pt, NiO/200Al_2_O_3_/Pt, and 50NiO/Al_2_O_3_/Pt is still higher than that of the single component Al_2_O_3_/Pt catalyst.

### Catalytic mechanism

Raman measurements were employed to characterize the used catalysts after reaction for 10 min, as shown in Fig. 2d. The Raman spectrum for the reference sample AB shows the B−N stretching mode at 727 and 783 cm^–1^, the B-H stretching mode at 2280 and 2375 cm^–1^, the N−H stretching mode at 3175, 3251, and 3316 cm^–1^, the BH_3_ deformation mode at 1159 and 1188 cm^–1^, and the NH_3_ deformation mode at 1600 cm^–1^, in agreement with the literature results^45^. For the Al_2_O_3_ support after reaction, these peaks can still be observed. For the NiO/Al_2_O_3_ and Al_2_O_3_/Pt samples after reaction, the NH_3_ deformation peak can be found at approximately 1600 cm^–1^, while the B−N and B-H peaks cannot be observed. These results demonstrate that NiO and Pt can readily dissociate the B−N and B−H bonds of AB in the presence of H_2_O.

**Fig. 3:**
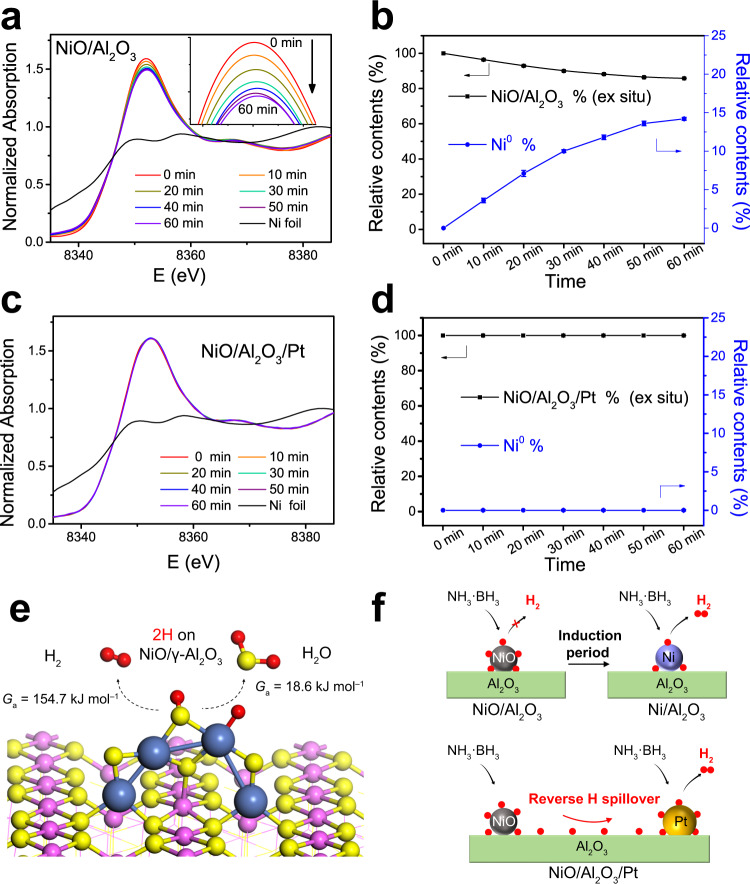
In situ XANES spectra for the catalysts and proposed mechanisms. In situ Ni K-edge XANES spectra for **a** NiO/Al_2_O_3_ and **c** NiO/Al_2_O_3_/Pt, and the percent of metallic Ni^0^ in the **b** NiO/Al_2_O_3_ and **d** NiO/Al_2_O_3_/Pt catalysts versus time during the reaction. The inset in **a** shows the expanded sections for the white line peaks. For each sample, the in situ spectrum is fitted by a linear combination of the ex situ spectrum and the spectrum for Ni foil. Error bars represent the fitting errors from XANES. **e** The free energy barriers (*G*_a_) at room temperature (298.15 K) for the formation and desorption of H_2_ (left arrow) and water (right arrow) on NiO/γ−Al_2_O_3_(100) computed by the DFT method. Colour legend: Al, pink; O, yellow; Ni, cyan; and H, red. **f** Proposed reaction mechanisms for NiO/Al_2_O_3_ and NiO/Al_2_O_3_/Pt in the H_2_ generation reaction. During the induction period for NiO/Al_2_O_3_, the H species generated at NiO sites are consumed to reduce the oxide catalyst to metallic Ni^0^. For NiO/Al_2_O_3_/Pt, the H species generated at NiO sites reversely spill across the Al_2_O_3_ support to Pt sites, where they can combine into H_2_ and release.

The dynamic behaviour of Ni species in the catalysts under the H_2_ generation reaction was probed with a quick XANES. The incident X-rays usually produce no damage to the material, as opposed to the action of electron or ion probes^46^. This capability of XANES makes it suitable for (in situ) catalyst structure studies^47^. From Fig. 3a, it can be found that the intensity of the white line peak for NiO/Al_2_O_3_ decreases with the reaction time, indicating that the Ni^2+^ species are gradually reduced. The Ni species are far from being fully reduced after reaction for 60 min. Furthermore, the in situ XANES spectrum was simulated by a linear combination of the ex situ spectrum of the as-prepared catalyst and the spectrum for the reference sample (Ni foil) to quantitatively reveal the dynamic behaviour of Ni species in the catalysts during the reaction. The experimental XANES spectra can be reproduced perfectly by simple linear fitting, with an extremely low R factor (Supplementary Fig. 12 and Supplementary Table 3). The reduction degrees for NiO/Al_2_O_3_ after reaction for 10, 20, 30, 40, 50, and 60 min are 3.6 ± 0.3, 7.1 ± 0.4, 10.0 ± 0.2, 11.8 ± 0.3, 13.6 ± 0.3, and 14.2 ± 0.2%, respectively (Fig. 3b). The reduction degree does not increase linearly with reaction time. These results demonstrate that metallic Ni^0^ species are generated gradually during the reaction, and the Ni^0^ generation rate slows down with time. For NiO/Al_2_O_3_/Pt, one may expect that more NiO will be reduced into metallic Ni^0^ after Pt addition due to spillover effects. Surprisingly, the in situ XANES spectra remain unchanged throughout the reaction, indicating that the reduction of Ni^2+^ species is totally inhibited after Pt addition (Fig. 3c, d).

## Discussion

Our results show that the H_2_ generation rate for Al_2_O_3_/Pt for the hydrolysis of AB can be enhanced after NiO addition, even though Pt and NiO are spatially separated by Al_2_O_3_ support. Here, the promotion functions of reverse spillover in the AB hydrolysis reaction are proven using a spatially separated catalyst as an ideal model catalyst based on kinetic analyses, Raman, in situ XANES, and DFT calculation results.

The Al_2_O_3_/Pt catalyst can catalyze the hydrolysis reaction of AB effectively, indicating that reactants can easily diffuse towards Pt sites. Pt nanoparticles for Al_2_O_3_/Pt are confined in the Al_2_O_3_ nanotubes. According to the BJH results, the average pore size of the nanotubes is larger than 50 nm, which is the main channel for the diffusion of reaction molecules to Pt sites. The Raman results demonstrate that Pt can readily dissociate the B−N and B−H bonds for AB in the presence of H_2_O, while the Al_2_O_3_ support cannot. Then, the generated H species are easily released from the Pt surface^48^.

The NiO/Al_2_O_3_ catalyst shows poor activity in the hydrolysis reaction. However, the Raman results demonstrate that NiO can also dissociate the B−N and B−H bonds of AB in the presence of H_2_O, generating H species. It is believed that NiO facilitates the adsorption of H−OH and the dissociation of electropositive H, which favours the attack of electronegative H in AB^18^. The H species generated at NiO sites can either be released as H_2_ from the oxide surface or can be consumed to reduce the NiO catalyst to metallic Ni^0^. The formation and desorption free energies for H_2_ and H_2_O on NiO sites in an aqueous environment were calculated using the DFT method (Fig. 3e and Supplementary Fig. 13). The free energy barrier (*G*_a_) at room temperature (298.15 K) for the formation of H_2_ is 154.7 kJ mol^–1^, whereas a much lower free energy barrier of 18.6 kJ mol^–1^ is required for H_2_O formation. This indicates that the H species tend to be consumed to reduce the NiO catalyst to form H_2_O. At the induction period for the H_2_ evolution curve for NiO/Al_2_O_3_, no H_2_ is produced, confirming that the H species are not released from the oxide surface. The in situ XANES results indicate that the generated H species are consumed for slowly reducing the oxide catalyst to metallic Ni^0^, which is consistent with the DFT results. After metallic Ni^0^ is produced, then the generated H species can be released from the metal surface (Fig. 3f).

For the spatially separated NiO/Al_2_O_3_/Pt catalyst, its H_2_ generation rate for the hydrolysis of AB is enhanced. The in situ XANES results indicate that no metallic Ni^0^ is observed in the NiO/Al_2_O_3_/Pt catalyst during the AB hydrolysis reaction, while reduction is observed in the NiO/Al_2_O_3_ catalyst. For both NiO/Al_2_O_3_ and NiO/Al_2_O_3_/Pt, NiO particles are located on the outer surfaces of Al_2_O_3_ nanotubes. N_2_ sorption isotherm results show that these two samples possess similar pore structures. Thus, the different reduction behaviours of NiO species for NiO/Al_2_O_3_ and NiO/Al_2_O_3_/Pt during the AB hydrolysis reactions are unlikely to be due to the mass transfer in the porous structures. The Raman results demonstrate that H species are generated at NiO and Pt sites at the same time. The H species generated at NiO sites can be released as H_2_ from the NiO surface, consumed to reduce NiO, or released from Pt sites through reverse spillover. Generally, the desorption of H_2_ molecules on Pt sites is quite easy and is considered to be barrier free^49,50^. Thus, reverse spillover is the lowest energy pathway. This is also confirmed experimentally. The H_2_ evolution curve for NiO/Al_2_O_3_ confirms that the H species are not released from NiO. The reduction of NiO is totally inhibited after Pt addition, revealing that the H species generated at NiO sites are not consumed for the reduction of NiO. The H species spill across the Al_2_O_3_ support from NiO to Pt sites, where they can combine into H_2_ and release (Fig. 3f). This is called the reverse spillover process, which accounts for the enhanced H_2_ generation rate for NiO/Al_2_O_3_/Pt after NiO addition.

**Fig. 4:**
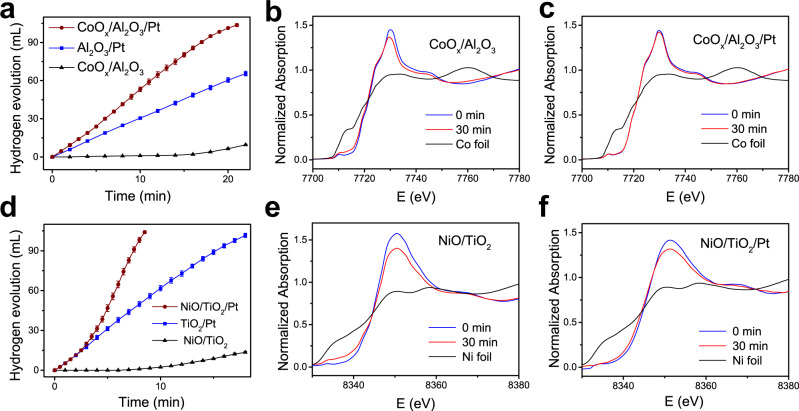
Reverse spillover effects in AB hydrolysis catalyzed by the CoO_*x*_/Al_2_O_3_/Pt and NiO/TiO_2_/Pt catalysts. **a** Volume of H_2_ generated from AB solution at 25 °C catalyzed by CoO_*x*_/Al_2_O_3_, Al_2_O_3_/Pt, and CoO_*x*_/Al_2_O_3_/Pt. In situ Co K-edge XANES spectra for **b** CoO_*x*_/Al_2_O_3_ and **c** CoO_*x*_/Al_2_O_3_/Pt catalysts before reaction and after reaction for 30 min and the spectrum for Co foil. **d** Volume of H_2_ generated from AB solution at 25 °C catalyzed by NiO/TiO_2_, TiO_2_/Pt, and NiO/TiO_2_/Pt. In situ Ni K-edge XANES spectra for **e** NiO/TiO_2_ and **f** NiO/TiO_2_/Pt catalysts before reaction and after reaction for 30 min and the spectrum obtained for Ni foil. Error bars correspond to the standard deviation for three independent measurements.

The reverse spillover phenomenon has also been confirmed in other catalytic systems, for example, in AB hydrolysis catalyzed by CoO_*x*_/Al_2_O_3_/Pt. As shown in Fig. 4a, an induction period can also be found in the H_2_ evolution curve for the CoO_*x*_/Al_2_O_3_ catalyst. For CoO_*x*_/Al_2_O_3_/Pt, a rapid and nearly linear H_2_ evolution curve is obtained, and its activity is higher than that of Al_2_O_3_/Pt. From the in situ XANES results, it can be found that after reaction for 30 min, the position of the white line peak for CoO_*x*_/Al_2_O_3_ shifts to a lower energy, and the intensity of the white line peak decreases, indicating that the Co oxide species are reduced (Fig. 4b). For CoO_*x*_/Al_2_O_3_/Pt, the change in the white line peak after the reaction is very slight (Fig. 4c). The in situ XANES spectrum was simulated by a linear combination of the ex situ spectrum for the as-prepared catalyst and the spectra obtained for the reference samples (CoO and metallic Co^0^) (Supplementary Fig. 14). For CoO_*x*_/Al_2_O_3_ after reaction for 30 min, 9.9% extra CoO and 12.9% extra metallic Co^0^ are formed. However, for CoO_*x*_/Al_2_O_3_/Pt, 9.7% of extra CoO and only 1.6% of extra metallic Co^0^ are formed. It can be concluded that the reduction of Co oxide species to metallic Co^0^ is mostly inhibited after Pt addition.

In addition to nonreducible Al_2_O_3_, when reducible TiO_2_ is used as a support, reverse spillover effects are also confirmed (Supplementary Figs. 15 and 16). As shown in Fig. 4d, the induction period in the H_2_ evolution curve for the NiO/TiO_2_ catalyst is shortened to 7 min, indicating that NiO supported on TiO_2_ is easier to reduce than that supported on Al_2_O_3_. The H_2_ evolution curve for NiO/TiO_2_/Pt is not linear. In the beginning, its rate is similar to that of TiO_2_/Pt. After that, the rate for NiO/TiO_2_/Pt begins to increase rapidly, exceeding the rate for TiO_2_/Pt. This implies that NiO sites are reduced to metallic Ni^0^ sites during the reaction. The in situ XANES spectra for NiO/TiO_2_ and NiO/TiO_2_/Pt are slightly rough, which is due to the high activities of the catalysts. The liquid reaction system is disturbed by a large amount of H_2_ bubbles, and thus, the X-ray absorption is affected. The in situ XANES (Fig. 4e, f) and its linear combination fitting results (Supplementary Fig. 17) demonstrate that for NiO/TiO_2_ after reaction for 30 min, 26.1% extra metallic Ni^0^ is formed, while for NiO/TiO_2_/Pt, 13.0% extra metallic Ni^0^ is formed. The reduction of NiO to metallic Ni^0^ is partially inhibited after Pt addition because of the reverse spillover effects. There are two competing pathways for the H species generated at the NiO sites of NiO/TiO_2_/Pt. A fraction of the H species spill over reversely to Pt sites; the rest is consumed to reduce NiO to metallic Ni^0^.

In summary, we designed spatially separated NiO/Al_2_O_3_/Pt catalysts to clarify the contribution of the reverse spillover effect to enhanced H_2_ generation rates. The in situ XANES results reveal that the H species generated at NiO sites are not consumed for the reduction of NiO to Ni^0^ or released as H_2_ at NiO sites. Instead, they reversely spill across the support to the Pt sites. The reverse spillover effects account for the enhanced H_2_ generation rates. The effects are also confirmed for CoO_*x*_/Al_2_O_3_/Pt and NiO/TiO_2_/Pt catalysts. In general, we believe that, with the help of an in-depth understanding of reverse spillover effects, this work can provide guidance for rationally designing highly efficient catalysts for H_2_ production in the future.

## Methods

### Synthesis of Al_2_O_3_/Pt catalysts

Typically, Pt nanoparticles (20 cycles) were deposited onto the CNC templates by ALD. Subsequently, the as-prepared Pt/CNCs were coated by an Al_2_O_3_ support film (50 cycles). Then, the CNC templates were removed by calcination at 500 °C for 1 h, obtaining an Al_2_O_3_/Pt catalyst.

### Synthesis of NiO/Al_2_O_3_/Pt and CoO_*x*_/Al_2_O_3_/Pt catalysts

To obtain NiO/Al_2_O_3_/Pt, NiO nanoparticles (100 cycles) were deposited onto Al_2_O_3_/Pt by ALD. The deposition cycles for NiO nanoparticles (50 and 100 cycles) and Al_2_O_3_ film (50, 100, and 200 cycles) can be adjusted, obtaining 50NiO/Al_2_O_3_/Pt, NiO/100Al_2_O_3_/Pt, and NiO/200Al_2_O_3_/Pt. To obtain CoO_*x*_/Al_2_O_3_/Pt, CoO_*x*_ nanoparticles (35 cycles) were deposited onto Al_2_O_3_/Pt by ALD.

### Synthesis of NiO/Al_2_O_3_ and CoO_*x*_/Al_2_O_3_ catalysts

CNCs were first coated with an Al_2_O_3_ layer (50 cycles) by ALD and then calcined at 500 °C for 1 h. Next, NiO (100 cycles) or CoO_*x*_ (35 cycles) nanoparticles were deposited by ALD, obtaining a NiO/Al_2_O_3_ or CoO_*x*_/Al_2_O_3_ catalyst.

### Synthesis of NiO/TiO_2_, TiO_2_/Pt, and NiO/TiO_2_/Pt catalysts

CNCs were first coated with a TiO_2_ layer (200 cycles) by ALD and calcined at 500 °C for 1 h. Then, NiO (100 cycles) nanoparticles were deposited by ALD, obtaining a NiO/TiO_2_ catalyst. To obtain TiO_2_/Pt, Pt nanoparticles (20 cycles) and a TiO_2_ film (200 cycles) were deposited onto a CNC template. Then, the CNC template was removed by calcination at 500 °C for 1 h, obtaining a TiO_2_/Pt catalyst. NiO (100 cycles) nanoparticles were deposited onto TiO_2_/Pt, producing NiO/TiO_2_/Pt.

### Sample characterization

The chemical compositions of these samples were determined by ICP-AES. The TEM and HRTEM images were taken on a JEOL-2100F microscope. The N_2_ sorption measurements were performed using Micromeritics Tristar 3000 at 77 K. XRD patterns were collected on a Bruker D8 Advance X-ray diffractometer using a Cu Kα source. XPS spectra were recorded on an AXIS ULTRA DLD spectrometer (Shimadzu/Kratos) to characterize the surface composition with the Al Kα line as the excitation source. H_2_-TPR experiments were performed using a tubular quartz reactor (TP-5080, Tianjin Xianquan, China), into which a 50 mg sample was loaded. The reduction was conducted in a 10% H_2_/N_2_ atmosphere at a heating rate of 10 °C/min. Hydrogen consumption was calculated by an external standard method using H_2_-TPR for CuO as the standard. The Raman spectra were performed on a LabRam HR Evolution (Horiba, France) spectrometer employing a He−Ne laser with an excitation wavelength of 532 nm. After the AB catalytic hydrolysis reaction for 10 min, the catalysts were centrifuged and dried in a vacuum oven at 30 °C. Finally, the samples were loaded, and the spectra were recorded at room temperature. The in situ XANES for Ni and Co K-edge were obtained on the 1W1B beamline of the Beijing Synchrotron Radiation Facility (BSRF), Institute of High Energy Physics, Chinese Academy of Sciences, and the BL14W1 and BL11B beamlines of the Shanghai Synchrotron Radiation Facility (SSRF), Shanghai Advanced Research Institute, Chinese Academy of Sciences. A Si (111) double-crystal monochromator was used to reduce the harmonic component of the monochrome beam. Ni and Co foil, NiO, CoO, and Co_3_O_4_ were used as reference samples and measured in transmission mode. The sample wafer was placed in the centre of a homemade in situ XANES cell. The spectra for the catalyst were first collected in transmission mode. After that, the AB solution (5 g L^–1^) was fed into the reactor at a speed of 5 mL min^–1^ by a sampling pump. The quick XANES were collected during the reaction at different times. IFEFFIT software was used to calibrate the energy scale, to correct the background signal, and to normalize the intensity. The spectra at the edge jump were simulated by a linear function of the reference Ni foil and the NiO-based catalyst before the reaction to estimate the proportion of metallic Ni^0^ in the catalyst during the reaction. The following formula was used:

(in situ XANES) = *f*_1_·(XANES of Ni foil) + *f*_2_·(ex situ XANES), where *f*_1_ and *f*_2_ are the fractions of the Ni foil and the as-prepared catalyst before the reaction, respectively.

### Catalytic testing

The catalytic performance of the samples was tested for AB hydrolytic dehydrogenation. Typically, the catalysts were first dispersed in deionized water (10 mL) placed in a round bottom flask with a magnetic stirrer at 25 ± 0.5 °C. The reaction was initialized by adding 48 mg of AB (Aldrich, 97%) into the reaction flask under stirring (700 rpm). A gas burette filled with water was connected to the flask to measure the amount of hydrogen evolved during the reaction by monitoring the displacement of the water level. In the AB concentration-dependent study, the reaction was performed at different AB concentrations (75, 112.5, 150, and 187.5 mmol L^–1^) at 25 ± 0.5 °C. To calculate the activation energy (*E*_a_), the reaction temperature was varied in the range of 20–35 °C, and the AB concentration was kept constant at 150 mmol L^–1^.

### Computational method

All DFT calculations were carried out using periodic spin-polarized density functional theory with the Perdew−Burke−Ernzerhof generalized gradient approximation functional^51^ as implemented in the Vienna ab initio simulation package (VASP)^52,53^. The calculations were performed using a plane-wave basis set, with a cut-off kinetic energy of 400 eV. Projector-augmented-wave^54^ potentials were used to describe the electron−ion interactions. Dispersion interactions were included by using the DFT-D3 (BJ) correction method of Grimme et al^55,56^. The crystal structure of γ-Al_2_O_3_ proposed by Gutiérrez et al.^57^ was adopted in our model system. The most stable (100) surface of γ-Al_2_O_3_ with three alumina layers and a Ni_4_O_4_ cluster adsorbed onto it were used for the NiO/γ-Al_2_O_3_(100) slab model. The two bottom layers of the slab were kept fixed. The thickness of the vacuum region was 20 Å. A Monkhorst-Pack grid was used for Brillouin-zone integrations with 1 × 1 × 1 k-mesh (gamma point) sampling. The solvation effect was included with an implicit solvation solvent of water using the VASPsol tool^58^. The free energies at room temperature (298.15 K) were obtained by adding to the DFT electronic energy (*E*), the zero-point energy, enthalpy, and entropy contribution from the vibrational modes. The transition states (TS) were calculated using the climbing image nudged elastic band method^59^, and frequency analysis was confirmed to verify the TS.

## Supplementary information

Supplementary Information

## Data Availability

The data that support the findings of this study are available from the corresponding authors upon reasonable request.
