# Supplementary material for: Enhanced hydrogen generation by reverse spillover effects over bicomponent catalysts
Source: Nat Commun. 2022 Jan 10;13:118. doi: 10.1038/s41467-021-27785-5 (PMC8748832; doi:10.1038/s41467-021-27785-5)
Supplement: Supplementary file 1 — Supplementary Information [file 41467_2021_27785_MOESM1_ESM.pdf]

Supplementary Information

Enhanced hydrogen generation by reverse spillover effects over bicomponent catalysts

Gao et al.

Supplementary Methods

Supplementary Figures 1-17

Supplementary Tables 1-3

Supplementary Methods

Synthesis of CNCs and the ALD process. The CNCs were synthesized by chemical vapor deposition using acetylene as a carbon source and copper nanoparticles as catalysts at 250 °C followed by a heat treatment at 900 °C in an Ar atmosphere for 2 h. Raw CNCs were refluxed in HNO_3 (15 wt %) for 4 h at 100 °C in an oil bath to remove the copper catalysts, then filtered and washed with deionized water and ethanol until there was no further change in pH (around pH = 7).

The ALD process was carried out in a hot-wall closed chamber-type ALD reactor. Prior to ALD, the CNCs (3 g) were dispersed in ethanol (100 mL) by ultrasonic agitation, and then 1 mL of the suspension was dropped onto a quartz wafer (10 cm×10 cm). After the samples were dried at ambient temperature, they were transferred to the ALD chamber. The Al_2O_3 film was deposited at 125 °C with trimethylaluminum (TMA) and deionized H_2O as precursors. The pulse, exposure, and purge times for the TMA were 0.02, 8, and 25 s, respectively, and for the H_2O, 0.1, 8, and 25 s, respectively. The TiO_2 film was deposited at 125 °C with titanium tetraisopropoxide (TTIP) and deionized H_2O as precursors. TTIP was maintained at 70 °C. The pulse, exposure, and purge times for the TTIP were 1, 8, and 25 s, respectively, and for the H_2O, 0.1, 8, and 25 s, respectively. Pt nanoparticles were deposited at 250 °C with trimethyl(methylcyclopentadienyl) platinum ($MeCpPtMe_3$) and ozone (O_3) as precursors. $MeCpPtMe_3$ was kept at 65 °C. The pulse, exposure, and purge times for the $MeCpPtMe_3$ were 0.5, 12, and 25 s, respectively, and for the O_3, 0.1, 12, and 25 s, respectively. NiO was deposited by sequential exposure of the samples to nickelocene ($NiCp_2$) and O_3 at 250 °C. $NiCp_2$ were kept at 75 °C. The pulse, exposure, and purge times for the $NiCp_2$ were 4, 5, and 20 s, respectively, and for the O_3, 0.05, 5, and 20 s, respectively. CoO_x nanoparticles were deposited at 250 °C with bis(cyclopentadienyl) cobalt (Cp_2Co) and O_3 as precursors. Cp_2Co was kept at 70 °C. The pulse, exposure, and purge times for the Cp_2Co were 5.5, 16, and 25 s, respectively, and for the O_3, 0.1, 12, and 25 s, respectively.

Catalyst characterizations. FIB based on high-brightness Ga liquid-metal ion sources was recorded in FIB-SEM instrument (Helios NanoLab 460HP). Typically, CNCs were first dispersed on a silicon wafer and then were deposited by Pt and Al_2O_3 ALD. After calcination, NiO particles were deposited. Then the Si wafer with the $NiO/Al_2O_3/Pt$ sample was transferred into the FIB system. A carbon layer was coated on the sample to enhance its stability. After that, a selected part of the protected sample was lifted out of the Si wafer and then mounted on a TEM grid. Finally, the sample was sliced down to ~100 nm along the vertical direction of the Al_2O_3 nanotubes by the Ga ion beam for TEM analysis. Aberration-corrected high-angle annular dark-field scanning transmission electron microscopy (AC-HAADF-STEM) was performed on a JEOL 2100F (FEI, Titan Cubed Themis G2 300) at 200 keV. High-sensitivity low-energy ion scattering spectra (HS-LEIS) were obtained using an Ion-TOF Qtac100 instrument. In order to improve the sensitivity of the analysis, Neon was selected as the ion source. In situ XANES experiment under H_2 atmosphere (0.6 MPa, 80 °C) were performed on the BL11B beamline of the Shanghai Synchrotron Radiation Facility (SSRF), Shanghai Advanced Research Institute, Chinese Academy of Sciences. The sample wafer was placed in the center of a self-made in-situ XANES cell. The spectra of the catalyst were first collected in the transmission mode at 80 °C. After that, H_2 atmosphere (0.6 MPa) were fed into the reactor. The quick XANES were collected during the reaction at different time. A Si (111) double-crystal monochromator was used to reduce the harmonic component of the monochrome beam. Ni foil and NiO were used as reference samples and measured in transmission mode. IFEFFIT software was used to calibrate the energy scale, to

correct the background signal and to normalize the intensity. The spectra at the edge jump were simulated by a linear function of the reference of Ni foil and as-prepared catalyst to estimate the proportion of metallic Ni^0 in the catalyst. The following formula was used:

(in situ XANES) = $f_1 \cdot$(XANES of Ni foil) + $f_2 \cdot$(ex situ XANES), where f_1 and f_2 are the fractions of the Ni foil and the as-prepared catalyst, respectively.

Supplementary Figures

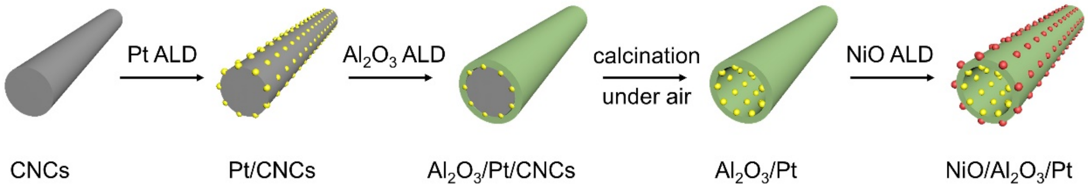

Supplementary Figure 1. Schematic illustration of the preparation process of NiO/Al_2O_3/Pt.

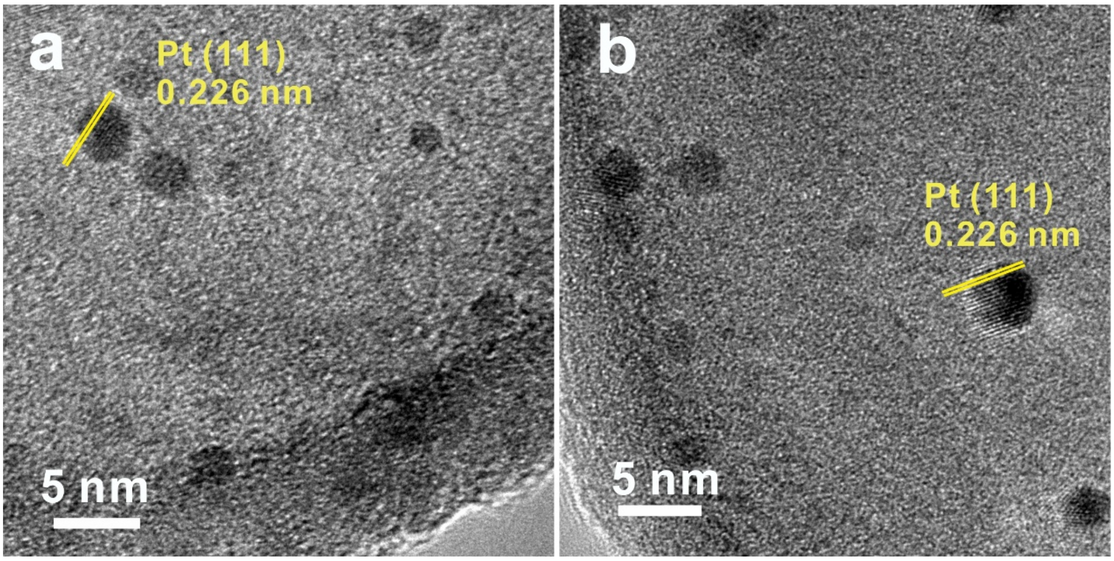

Supplementary Figure 2. Structural characterization of the catalysts. HRTEM images of (a) $NiO/Al_2O_3/Pt$ and (b) Al_2O_3/Pt.

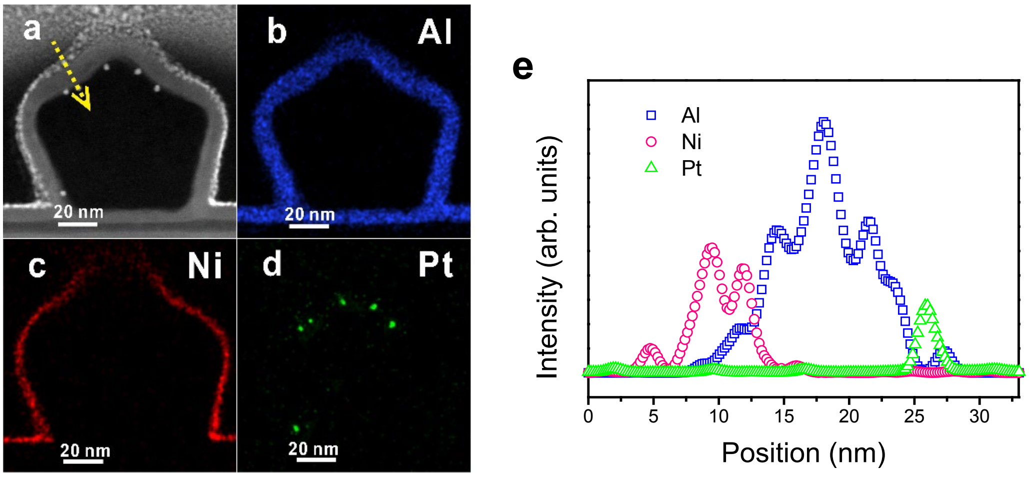

Supplementary Figure 3. Structural characterization of NiO/Al_2O_3/Pt. (a) STEM image and (b–d) EDX elemental mappings of a cross-sectional specimen of NiO/Al_2O_3/Pt prepared by focused ion beam milling. (e) Compositional point profile of Al, Ni, and Pt from the specimen recorded along the yellow arrow shown in a.

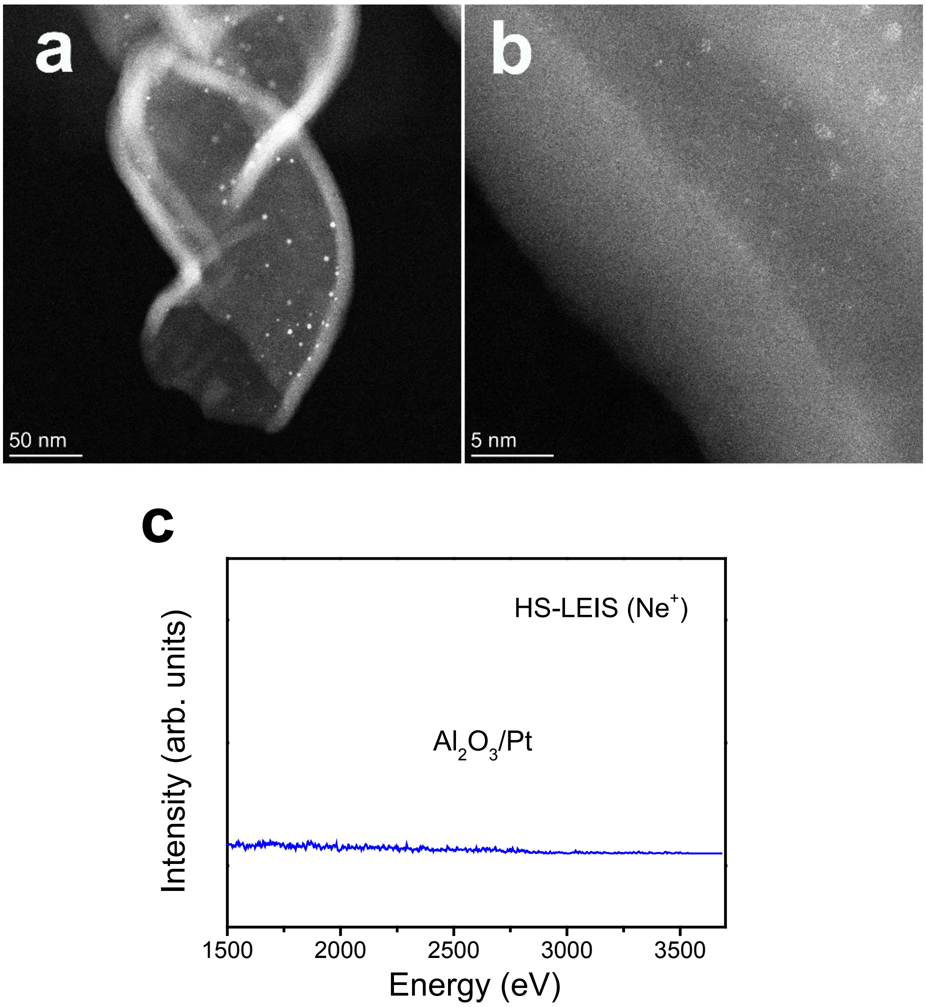

Supplementary Figure 4. (a and b) AC-HAADF-STEM images and (c) HS-LEIS spectrum for Al_2O_3/Pt. From Supplementary Figure 4a and b, it can be seen that besides Pt nanoparticles, Pt single atoms and Pt clusters are also confined in the Al_2O_3 nanotubes. However, no Pt particles are observed on the outer surfaces of Al_2O_3 nanotubes. Furthermore, HS-LEIS was also performed. No peak is found around 3300 eV (signal of Pt) for Al_2O_3/Pt (Supplementary Figure 4c). All the results demonstrate that for the Al_2O_3/Pt sample, Pt particles do not move to the outer surface of Al_2O_3 nanotubes.

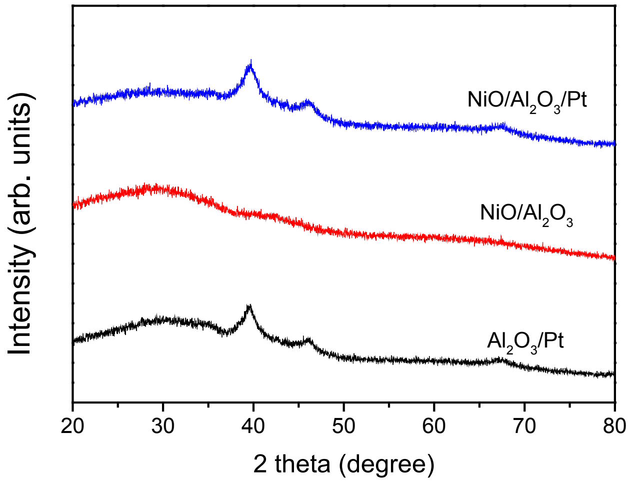

Supplementary Figure 5. XRD patterns of different catalysts. For Al_2O_3/Pt and NiO/Al_2O_3/Pt catalysts, four diffraction peaks at around 39.7°, 46.2°, 67.5° and 81.3° are observed, which can be ascribed to (111), (200), (220) and (311) crystal planes of face-centered cubic Pt^0 (JCPDS No.65-2868), respectively.

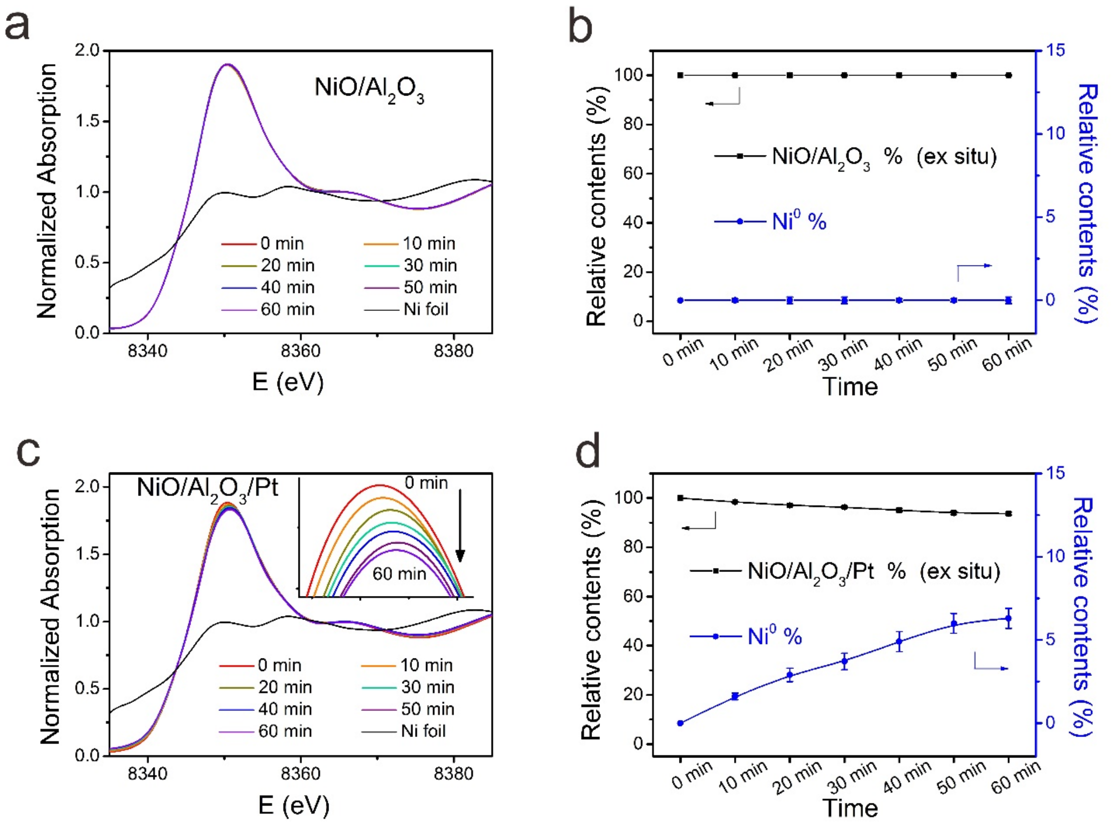

Supplementary Figure 6. In situ Ni K-edge XANES spectra of (a) NiO/Al_2O_3 and (c) $NiO/Al_2O_3/Pt$ under H_2 atmosphere (0.6 MPa, 80 °C), and the percent of metallic Ni^0 in the (b) NiO/Al_2O_3 and (d) $NiO/Al_2O_3/Pt$ catalysts versus time. Inset of Fig. 6c shows the expanded sections of white line peaks. For each sample, the in situ spectrum is fitted by a linear combination of the ex situ spectrum and the spectrum of Ni foil. Error bars represent the fitting errors from XANES.

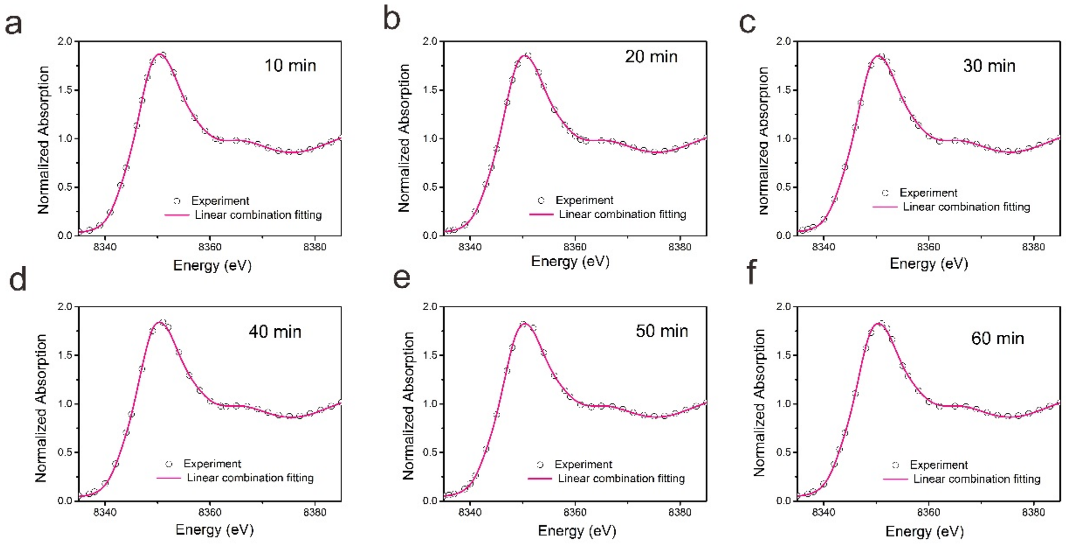

Supplementary Figure 7. Linear combination fitting of the in situ XANES spectra for NiO/Al_2O_3/Pt under H_2 atmosphere (0.6 MPa, 80 °C) in (a) 10 min, (b) 20 min, (c) 30 min, (d) 40 min, (e) 50 min, and (f) 60 min.

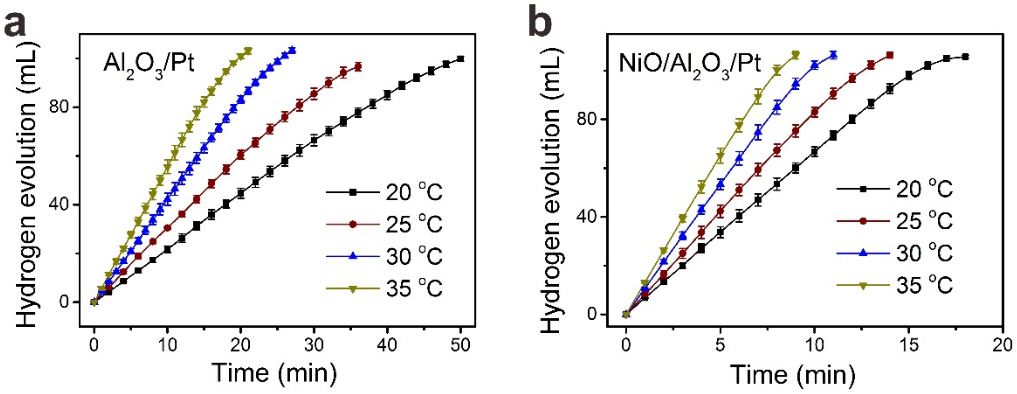

Supplementary Figure 8. Kinetic experiments of Al_2O_3/Pt and NiO/Al_2O_3/Pt catalysts. Volume of H_2 generated from AB solution (0.15 mol L^{-1}) catalyzed by (a) Al_2O_3/Pt and (b) NiO/Al_2O_3/Pt at different temperatures. Error bars correspond to the standard deviation of three independent measurements.

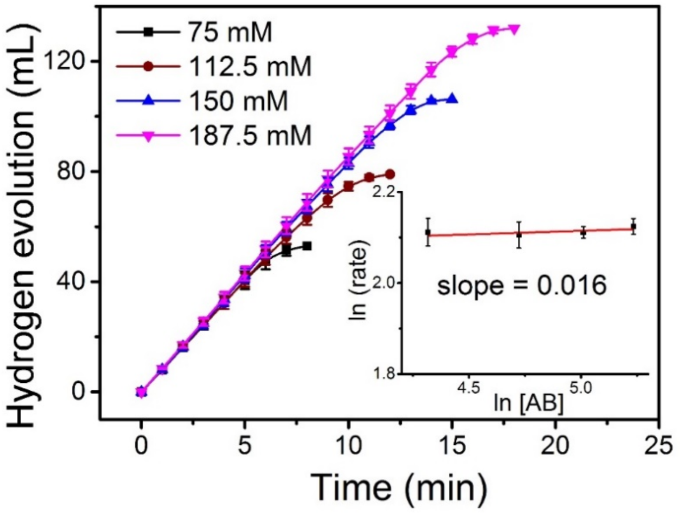

Supplementary Figure 9. Kinetic experiments of the NiO/Al_2O_3/Pt catalyst. Volume of H_2 generated versus time at 25 ^oC catalyzed by NiO/Al_2O_3/Pt with different AB concentration. Error bars correspond to the standard deviation of three independent measurements.

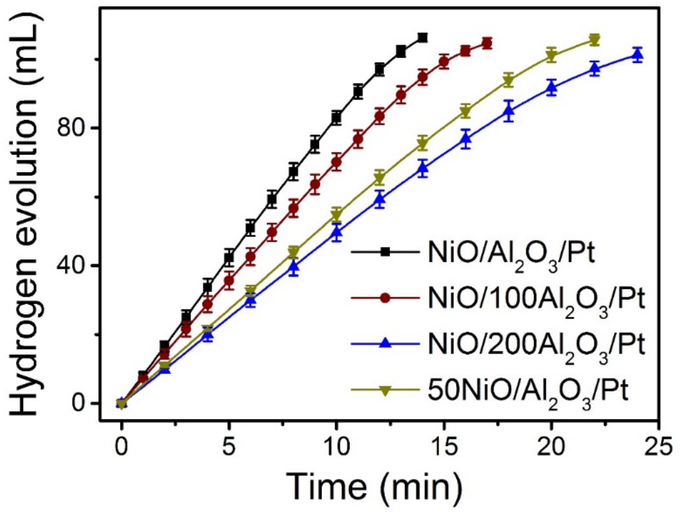

Supplementary Figure 10. Catalytic performances of the catalysts for the dehydrogenation reaction of AB. Volume of H_2 generated versus time at 25 ºC catalyzed by $NiO/Al_2O_3/Pt$, $NiO/100Al_2O_3/Pt$, $NiO/200Al_2O_3/Pt$, and $50NiO/Al_2O_3/Pt$. Error bars correspond to the standard deviation of three independent measurements.

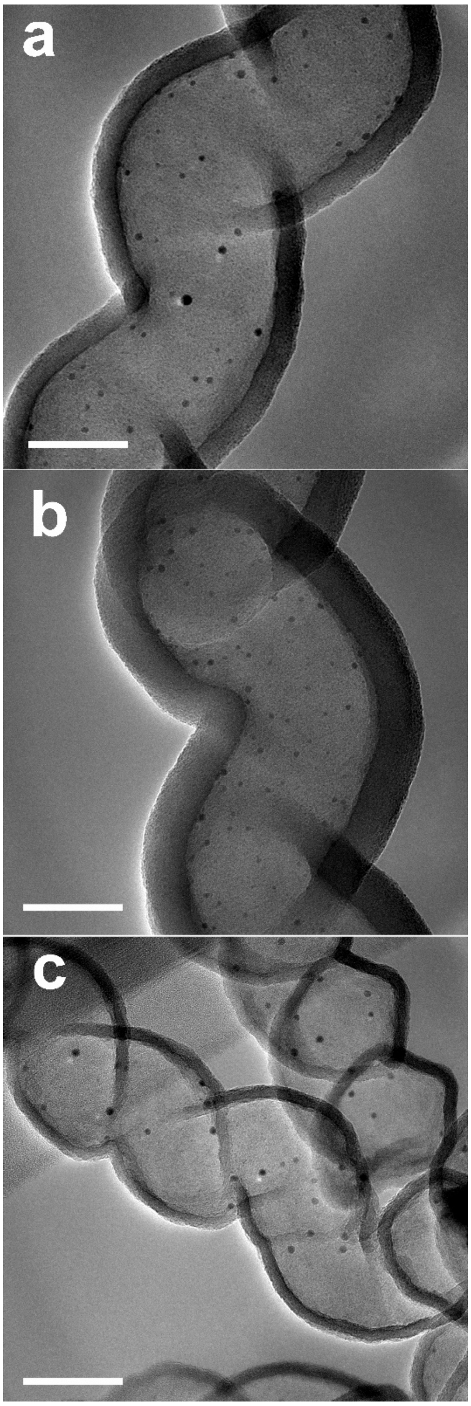

Supplementary Figure 11. Structural characterization of the catalysts. TEM images of (a) NiO/100Al_2O_3/Pt, (b) NiO/200Al_2O_3/Pt, and (c) 50NiO/Al_2O_3/Pt (scale bar, 50 nm).

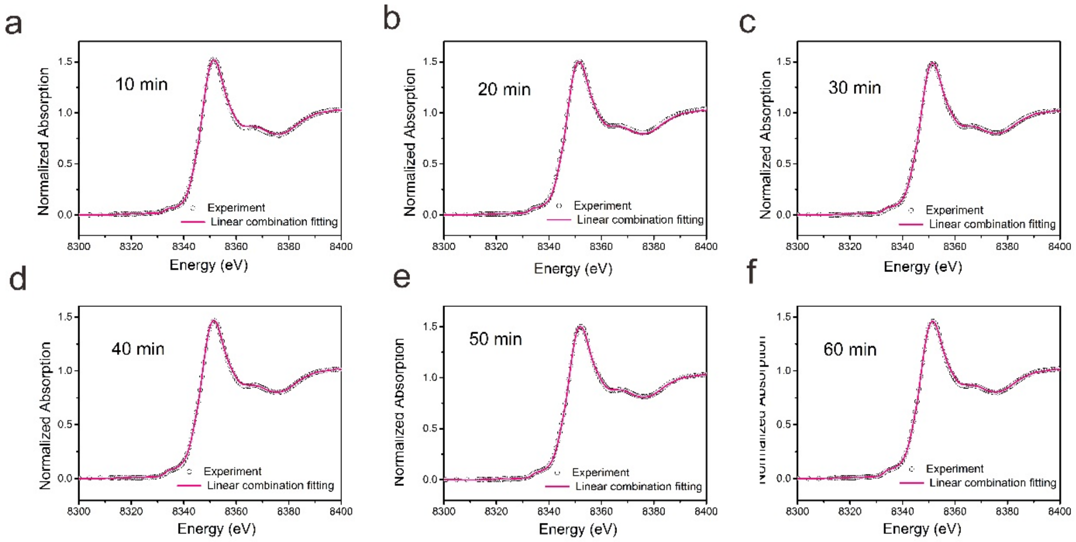

Supplementary Figure 12. Linear combination fitting of the in situ XANES spectra for NiO/Al_2O_3 under H_2 generation reaction after reaction for (a) 10 min, (b) 20 min, (c) 30 min, (d) 40 min, (e) 50 min, and (f) 60 min. The in situ XANES spectra were simulated by a linear combination of the ex-situ spectrum of the as-prepared catalyst and the spectra of reference sample (Ni foil) to quantitatively reveal the dynamic behavior of Ni species in the catalysts during the H_2 generation reaction.

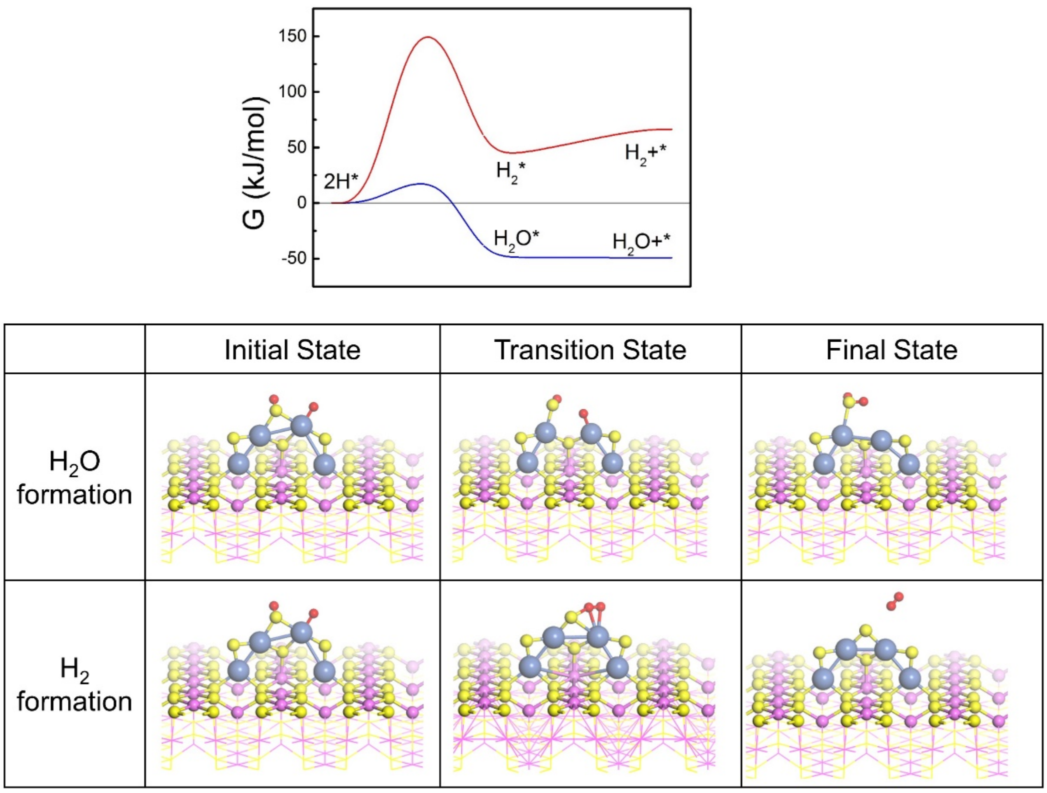

Supplementary Figure 13. The transition state (TS) of the formation of H_2 and H_2O on NiO/γ-$Al_2O_3(100)$. The TS were calculated using the climbing image Nudged Elastic Band (NEB) method, and frequency analysis was confirmed to verify the TS.

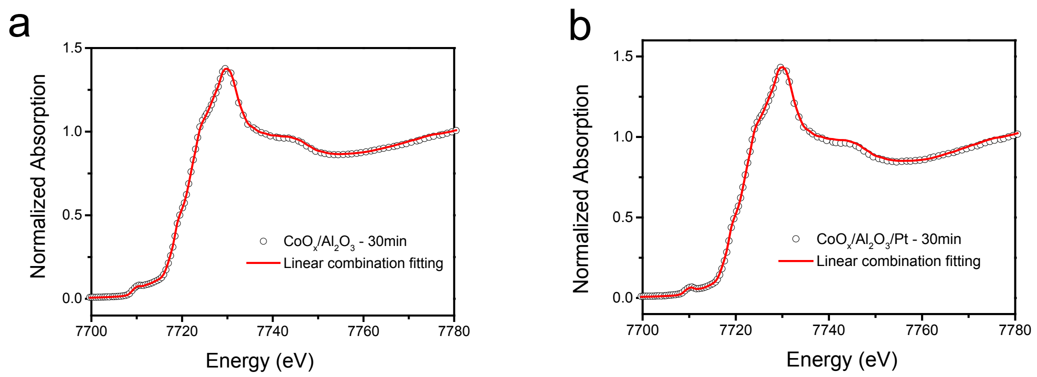

Supplementary Figure 14. Linear combination fitting of the in situ XANES spectra for (a) CoO_x/Al_2O_3 and (b) CoO_x/Al_2O_3/Pt under H_2 generation reactions. The in situ XANES spectrum is simulated by a linear combination of the ex-situ spectrum of the as-prepared catalyst and the spectra of reference samples (CoO and metallic Co^0).

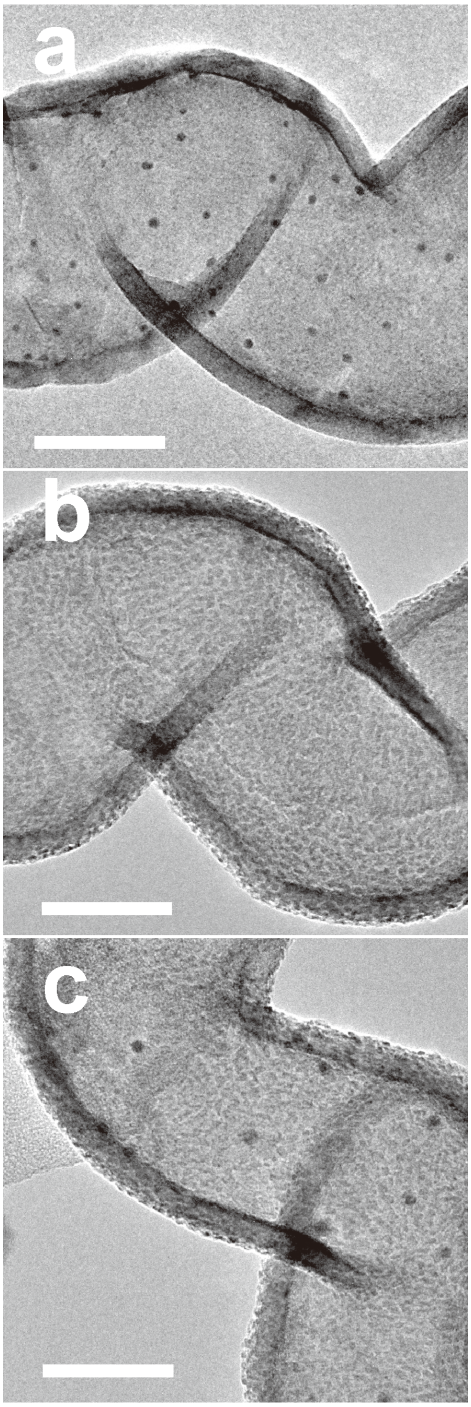

Supplementary Figure 15. Structural characterization of the catalysts. TEM images of (a) TiO_2/Pt, (b) NiO/TiO_2, and (c) NiO/TiO_2/Pt (scale bar, 50 nm).

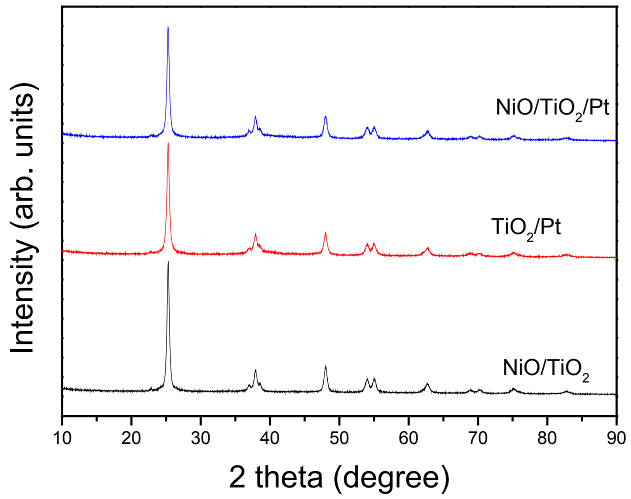

Supplementary Figure 16. XRD patterns of different catalysts. A strong peak at around 25.2° and several weak peaks can be observed, which can be well indexed to anatase TiO_2 (JCPDS No.21 1272). No obvious peak assigned to Pt or NiO is detected, which is due to the high dispersion of ALD prepared nanoparticles.

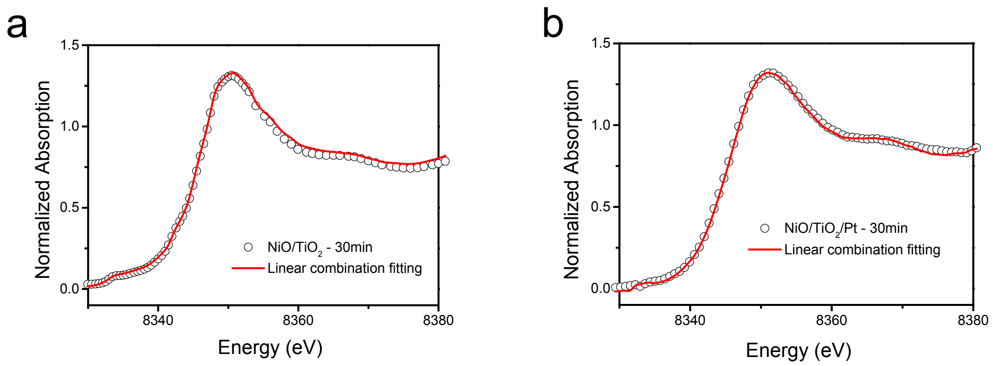

Supplementary Figure 17. Linear combination fitting of the in situ XANES spectra for (a) NiO/TiO_2 and (b) $NiO/TiO_2/Pt$ under H_2 generation reactions.

Supplementary Tables

Supplementary Table 1. The reduction peak areas and the quantification results of the H_2-TPR curves.

Catalysts	Reduction peak temperature (°C)	Peak area	Total area	Total consumed H_2 (mmol H_2 g^{-1})
Al_2O_3/Pt	385	1152.2	1152.2	0.31
NiO/Al_2O_3	349	56.9	4792.1	1.29
	431	4735.2		
NiO/Al_2O_3/Pt	326	1592.6	6928.5	1.86
	408	5335.9		

Supplementary Table 2. Linear combination fitting results of in situ XANES spectra for $NiO/Al_2O_3/Pt$ under H_2 atmosphere (0.6 MPa, 80 ^oC).

time (min)	10	20	30	40	50	60
Ni^0 percent (%)	1.6 ± 0.2	2.9 ± 0.4	3.7 ± 0.5	4.9± 0.6	6.0 ± 0.6	6.3 ± 0.6
R-factor	0.0000349	0.0002075	0.0002821	0.0003661	0.0003355	0.0003693

Supplementary Table 3. Linear combination fitting results of in situ XANES spectra for NiO/Al_2O_3 under H_2 generation reactions (AB solution).

Reaction time (min)	10	20	30	40	50	60
Ni^0 percent (%)	3.6 ± 0.3	7.1 ± 0.4	10.0 ± 0.2	11.8 ± 0.3	13.6 ± 0.3	14.2 ± 0.2
R-factor	0.000069	0.000139	0.000058	0.000093	0.000076	0.000057
